# Fertility of Spiny-Cheek Crayfish (*Orconectes limosus* Raf.) from the Vistula Lagoon

**DOI:** 10.1007/s00128-019-02543-y

**Published:** 2019-01-17

**Authors:** Radomir Graczyk, Bogusław Chachaj, Magdalena Stanek, Janusz Dąbrowski, Grzegorz Gackowski

**Affiliations:** 1 Department of Animal Biology and Environment, Faculty of Animal Breeding and Biology, UTP University of Science and Technology, Hetmańska St. 33, 85-039 Bydgoszcz, Poland; 2 Department of Animal Physiology, Physiotherapy and Nutrition, Faculty of Animal Breeding and Biology, UTP University of Science and Technology, Mazowiecka St. 28, 85-084 Bydgoszcz, Poland

**Keywords:** Spiny-cheek crayfish, Absolute fertility, Relative fertility, The Vistula Lagoon

## Abstract

The aim of this study was to examine fertility of spiny-cheek crayfish harvested in the first half of April from the Vistula Lagoon and to compare it with that of the crayfish from freshwater habitats. The sample consisted of 47 sexually mature females shortly before they were ready to lay eggs. After determining the absolute fertility (number of eggs per ovary), the relative fertility was calculated (number of eggs per 1 g of body weight). Absolute and relative fertility of spiny-cheek crayfish females with total body length 8.1–11.6 cm was 535 and 17 eggs, respectively. Absolute and relative fertility was correlated with total body length and weight. Along with the increase in these parameters, the absolute fertility increased and the relative fertility decreased. A comparison of absolute and relative fertility of spiny-cheek crayfish from the Vistula Lagoon with the representatives of this species from freshwater habitats such as the Brda River and the Lake Dgał Wielki, showed no significant differences.

The tradition of preparing crayfish meat in Poland has a long history and it may not be a commonly known fact, that it goes back even to mediaeval times (Mastyński and Andrzejewski 2005). Despite the fact that meat yield in these animals is not high due to their characteristic body structure, among many consumers this product is considered a culinary delicacy which could be compared with caviar. Spiny-cheek crayfish (*Orconectes limosus* Raf.) was brought to Europe from the North America at the end of the nineteenth century. This species can live in a variety of freshwater habitats but also in slightly salty waters (Paavola et al. 2005; Holdich and Black 2007; Pârvulescu et al. 2009; Jaszczołt and Szaniawska 2011; Aklehnovich and Razlutskij 2013). Spiny-cheek crayfish currently populates almost entire Poland, except for its south-east areas (Strużyński and Śmietana 1998; Krzywosz 2004; Grabowski et al. 2005). It is so widespread due to its no limited environmental requirements, effective reproduction and short period of embryonic development (Kossakowski 1966; Kozák et al. 2006; Buřič et al. 2013). This species is invasive to native ones (the noble crayfish (*Astacus astacus*) or the Danube crayfish (*Astacus leptodactylus*) and thus it is not a protected species (Krzywosz et al. 1995; Pyka and Kraśniewski 1997; Strużyński and Śmietana 1998; Śmietana 2000). In Poland, spiny-cheek crayfish is the only species capable of living in polluted and eutrophic aquatic habitats (Strużyński 1994; Śmietana and Strużyński 1999), including the Vistula Lagoon.

The aim of this study was to investigate fertility of spiny-cheek crayfish from the Vistula Lagoon and to compare it with that of the crayfish from freshwater reservoirs such as the Brda River and the Lake Dgał Wielki). Because it is a dominant species, research on its reproduction and the quality of its meat are reasonable. Polish research literature comprises a few works on fertility of spiny-cheek crayfish inhabiting several lakes (Stypińska 1972b, 1973; Chybowski 2007) and the Brda River (Gackowski et al. 2007). The research conducted by Stanek et al. (2017) concerning spiny-cheek crayfish from the Lake Gopło indicated that concentration of toxic metals in analyzed crayfish meat did not exceed acceptable values from the regulation of the EU (2011). The meat of this crayfish was reach in fatty acids from the PUFA group (48%–49% proportion of all acids), low percentage content of fat (1%–1.35%), and a small amount of cholesterol which ranged from 86.96 to 96.68 mg·100 g^−1^ (Stanek et al. 2013).

## Materials and Methods

The area of the Vistula Lagoon is 838 km^2^, of which 328 km^2^ belong to Poland. The lagoon length is about 90.7 km, of which 35.1 km is located in Poland. The reservoir width ranges from 6.8 to 13 km, with an average of 9.2 km. The average depth of the Polish part of the lagoon is 2.4 m, and maximum is 4.4 m. The Vistula Lagoon and the Baltic Sea are connected via a narrow canal located in the Russian part of the reservoir (Strait of Baltiysk). During heavy storms marine waters enter the lagoon through the canal. Average water salinity in the lagoon is 3.0‰. In the spring the salinity is low and it increases at the end of the summer. Polish section of a direct catchment of the lagoon comprises mainly arable lands (64%) and forests (18.3%). Ecological status of the water is unsatisfactory due to the quality of biological parameters (chlorophyll a, macrobentos) and physical and chemical factors (transparency, saturation with oxygen, pH and total nitrogen). The main problem of this reservoir is eutrophication caused by the supply of biogenic substances from multiple point and diffuse sources and rivers. This leads to abundant summer phytoplankton blooms, including blue-green algae (Kopiec 2014).

**Fig. 1:**
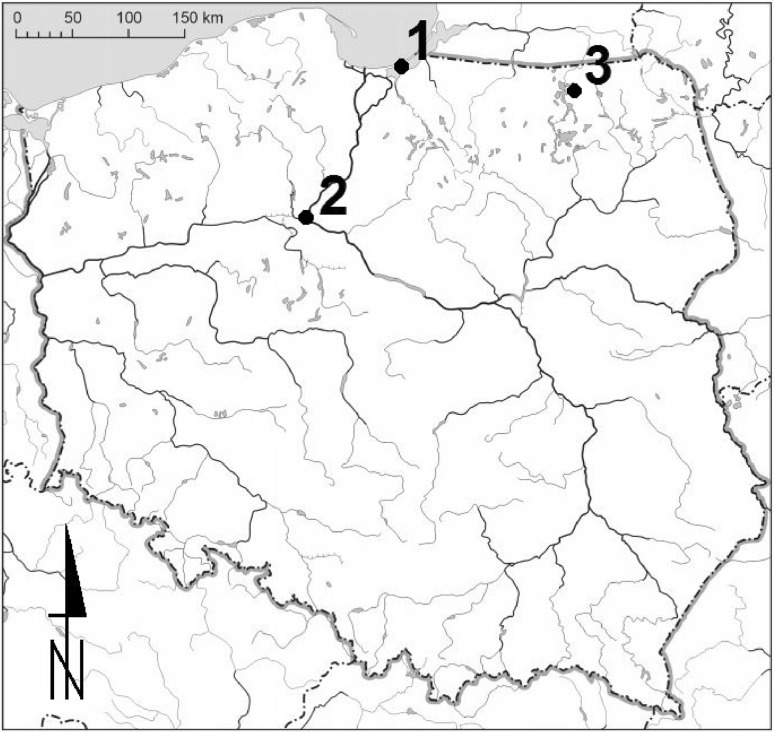
Location of spiny-cheek crayfish harvesting spot in the Vistula Lagoon (*1*) and two places from crayfish were caught by Gackowski et al. 2007 (*2*—Brda River) and by Chybowski 2007 (*3*—Lake Dgał Wielkie)

Spiny-cheek crayfish were harvested from the Vistula Lagoon, near Krynica Morska (location 1., Fig. 1) in the first half of April 2016. The biological material consisted of 47 sexually mature females, which were shortly before laying eggs. Total body length (from the tip of the rostrum to the rear edge of the telson) of each individual was measured, with an accuracy of 0.1 mm. Body mass was determined with an accuracy of 0.1 g. The group of examined crayfish was divided into four groups related with the weight and length. The samples of gonads were preserved in 4% solution of formalin. After that, the absolute fertility (number of eggs in an ovary) and the relative fertility (number of eggs per 1 g of body weight) were calculated. Next, the female fertility was analyzed by the assumed classes of total length and weight.

Data analyses were performed using MS Excel 2007 spreadsheet and Statistica 12.0 package (StatSoft 2012), according to the methods of statistical inference (Kala 2005; Stanisz 2006a, b). Basic descriptive statistics included means ($\overline {{\text{x}}}$), standard deviation (SD), and minimum (Min) and maximum values (Max). Normality of the distribution was tested with Kolmogorov–Smirnov test and homogeneity of variance was tested using Levene’s test. One-way analysis of variance (ANOVA) was performed to determine the significance of differences between the means of fertility-related values. Sperman correlation coefficient was used (Bolboacă and Jäntschi 2006) to measure the relationship between total length, body weight and absolute and relative fertility. The correlation coefficient was interpreted as described by Stanisz (2006a). The level of significance assumed for all statistical tests was α = 0.05.

## Results and Discussion

Total length of the examined females ranged from 8.1 to 11.6 cm, with an average of 10.2 cm. Body weight ranged from 14.5 to 47.0 g, with an average of 32.5 g. Absolute fertility of the females from the Vistula Lagoon ranged from 295 to 862 eggs, with an average of 535.5 eggs. Significant differences in absolute fertility between shorter and longer, and lighter and heavier individuals were observed (Tables 1, 2). The coefficient of variation reached markedly higher value (about 20%) in crayfish with greater length and weight as compared with the individuals with lesser length and weight.

**Table 1:** Fertility of spiny-cheek crayfish from the Vistula Lagoon across total length groups

Total length (cm)	n	Absolute fertility			Relative fertility		
		Range	Mean ± SD	Vzm	Range	Mean ± SD	Vzm
8.1– 9.0	5	295–388	358.0^a^ ± 38.5	10.7	16.4–24.7	20.7^a^ ± 3.4	16.5
9.1–10.0	11	385–599	494.3^ab^ ± 72.91	14.8	15.9–21.6	18.7^ab^ ± 2.0	10.7
10.1–11.0	21	366–802	557.2^bc^ ± 115.8	20.8	10.4–21.4	16.3^bc^ ± 3.4	20.7
11.1–12.0	10	452–862	624.0^c^ ± 117.4	18.8	10.5–18.3	14.6^c^ ± 2.3	16.0

^a, b, c^Differences between study classes; mean values with the same letter are not significantly different, at *p* ≤ 0.05; absolute fecundity (*F* = 8.40 *p* = 0.001), relative fecundity (*F* = 6.61 *p* = 0.001)

**Table 2:** Fertility of spiny-cheek crayfish from the Vistula Lagoon across body weight groups

Body weight (g)	n	Absolute fertility			Relative fertility		
		Range	Mean ± SD	Vzm	Range	Mean ± SD	Vzm
10.1–20.0	3	295–388	344.3^a^ ± 46.8	13.6	20.3–24.7	22.8^a^ ± 2.2	9.8
20.1–30.0	13	370–599	475.5^ab^ ± 78.7	16.5	15.9–21.6	18.6^ab^ ± 1.9	10.4
30.1–40.0	22	366–802	557.6^bc^ ± 112.9	20.3	10.4–21.4	16.1^bc^ ± 3.2	20.1
40.1–50.0	9	452–862	631.8^c^ ± 121.8	19.3	10.5–18.3	14.6^c^ ± 2.5	16.9

^a, b, c^Differences between study classes; mean values with the same letter are not significantly different, at *p* ≤ 0.05; absolute fecundity (*F* = 7.72*p* = 0.000), relative fecundity (*F* = 9.13*p* = 0.000)

**Fig. 2:**
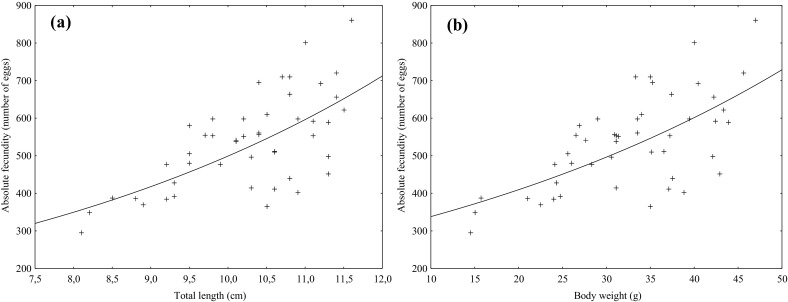
Relationship between total length (cm) and body weight (g) and absolute fertility of spiny-cheek crayfish caught from the Vistula Lagoon

Absolute fertility of the females crayfish was expressed by the number of eggs in the ovaries during their full maturity. An analysis of absolute fertility in four length and weight related groups revealed that longer and heavier females produced greater average number of eggs per ovary (Tables 1, 2). This was proved by statistically significant differences between average values of absolute fertility in the assumed groups related with length and weight. Positive and statistically significant correlation was found between total length and absolute fertility (r(X, Y) = 0.65) and between body weight and absolute fertility (r(X, Y) = 0.64) (Fig. 2).

**Fig. 3:**
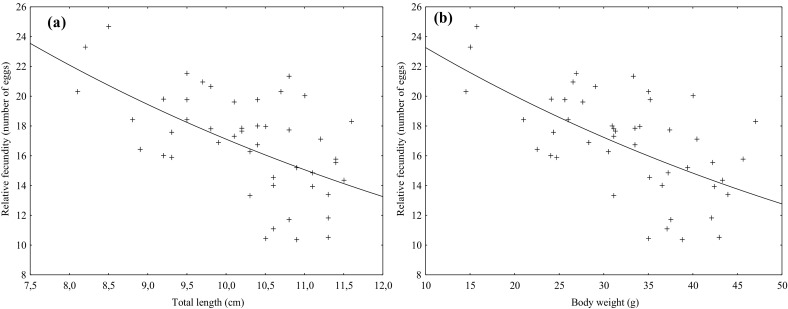
Relationship between body weight (g) and body weight (g) and relative fertility of spiny-cheek crayfish caught from the Vistula Lagoon

Relative fertility (per 1 g of female body weight) of spiny-cheek crayfish ranged from 10 to 25 eggs, with an average of 16.9 eggs. In separate length and weight related group, substantial differences in relative fertility, especially in longer and heavier individuals, were observed (Tables 1, 2). The greatest variability in the relative fertility was noticed in third group related with length (10.1 to 11.0 cm) and in third group related with weight (30.1 to 40.0 g). The coefficient of variation for these classes reached about 20%. An analysis of relative fertility in length and weight related groups showed that the longer and heavier females were, the smaller number of eggs they produced (Tables 1, 2). This was confirmed by statistically significant differences between average values of relative fertility in the assumed groups of length and weight. Moreover, negative and statistically significant correlation was found between total length and relative fertility (r(X,Y) = − 0.57) and between body weight and relative fertility (r(X,Y) = − 0.60) (Fig. 3).

**Fig. 4:**
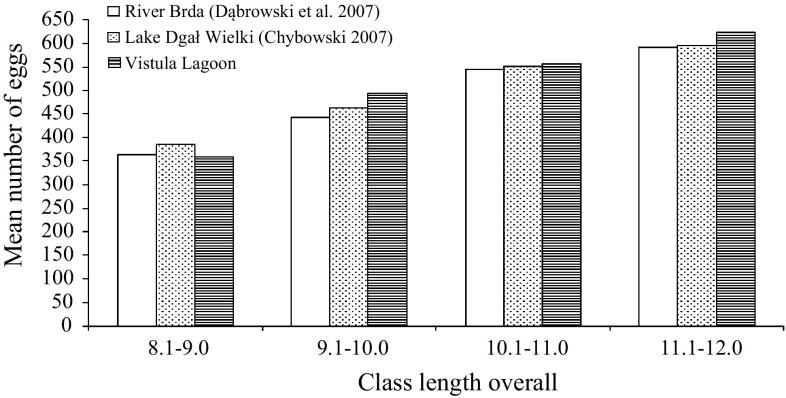
Absolute fertility of spiny-cheek crayfish collected from different reservoirs across four total body length related groups

Absolute fertility of spiny-cheek crayfish from the Vistula Lagoon and other reservoirs across their total body length are presented in Fig. 4.

According to Pieplow (1938) and Müller (1973) spiny-cheek crayfish reaches sexual maturity when its total length is 6 cm. Contrary to that, Kossakowski (1961) observed that the females from the Lake Wdzydze reached sexual maturity at 5 to 6 cm. In Poland, spiny-cheek crayfish mating occurs in the autumn. However, in some lakes they were observed to mate in the spring, usually in mid-May (Ulikowski and Borkowska 1999). Spiny-cheek crayfish reproduces in both: freshwater and slightly salty water. In laboratory tests, Jaszczołt and Szaniawska (2011) demonstrated that spiny-cheek crayfish may reproduce and thrive at the maximum salinity of 7 PSU. This value corresponds approximately to the average salinity of the Baltic Sea surface water. Spiny cheek crayfish is a common species and undoubtedly the most represented species of crayfish in the waters of Pomerania, Wielkopolska, Warmia and Mazury and Masovia. This invasive species is currently found in all types of freshwater reservoirs all over Poland (Aklehnovich and Razlutskij 2013; Protasowicki et al. 2013; Kouba et al. 2014).

Absolute fertility of female crayfish, expressed by the number of eggs in the ovaries during their full maturity, in native species (*A. astacus* and *A. leptodactylus*) does not always result in a reproductive success. However, for spiny-cheek crayfish absolute fertility is more important. Developing eggs located under abdomen are exposed to numerous risks. The period over which the eggs develop and reside on the abdomen is about 6 months for native species and only 5–6 weeks for spiny-cheek crayfish (Kossakowski 1966; Krzywosz 1999).

Strong correlation between total length and absolute fertility (r(X, Y) = 0.65) and between body weight and absolute fertility (r(X, Y) = 0.64) was found in crayfish from the Vistula Lagoon. Positive and significant correlations between total length, body weight and absolute fertility had been earlier observed in spiny-cheek crayfish from the Lake Wdzydze (Stypińska 1972b) and the Lake Dgał Wielki (Masurian Lake Discrict) (Chybowski 2007), Kořensko Reservoir (Kozák et al. 2006) and the Brda River (Gackowski et al. 2007). Statistically significant positive correlation between total length and absolute fertility were also reported for other species of crayfish (Penn 1943; Stypińska 1972a, 1973; Chybowski 2013).

A comparison of mean absolute fertility in spiny-cheek crayfish from the Vistula Lagoon and from the Brda River and the Lake Dgał Wielki revealed that the crayfish from the Vistula Lagoon with greater total body length produced slightly higher number of eggs but differences were not statistically significant (Fig. 4). Stypińska (1972b) reported on average absolute fertility of 375 to 440 eggs in the length classes of 8.5–9.4 cm and 9.5–10.4 cm for spiny-cheek crayfish from the Lake Wdzydze. Moreover, in greater length classes absolute fertility was slightly lower as compared with the females from the Vistula Lagoon.

Negative and statistically significant correlations between total length, body weight and relative fertility of spiny-cheek crayfish from the Vistula Lagoon were found. A decline in relative fertility along with increasing body length was also observed in spiny-cheek crayfish from the Lake Wdzydze (Stypińska 1972b) and from the Brda River (Gackowski et al. 2007).

Relative and absolute fertility of the investigated crayfish collected from the Vistula Lagoon was similar to that of spiny-cheek crayfish collected from the other freshwater reservoirs, regardless of their geographical location, the degree of water salinity or the degree of eutrophication. Recent analyses of the Vistula Lagoon waters carried out in 2014 confirmed that the ecological status of this waters was assessed as poor due to the quality of biological elements and no exceedances of substances particularly harmful to the environment were found. Report elaborated by the Voivodeship Inspectorate for Environmental Protection in Elbląg showed that the main problem of the Vistula Lagoon waters is eutrophication caused by supplying waters with biogenic substances supplied by rivers (Report 2015). Lake Dgał Wielkie is a mesotrophic and flow-through lake located in the Masurian Lake District (location 3., Fig. 1). Spiny cheek crayfish research conducted by Protasowicki et al. (2013) showed that the concentrations of toxic metals (Hg, Pb, Cd) in the abdominal muscles were several fold lower than the maximum permissible levels (EC, 2011). Report elaborated by the Voivodeship Inspectorate for Environmental Protection in Bydgoszcz (Report 2016), it follows that due to physical and chemical conditions, the state of the waters of the Brda River (location 2., Fig. 1) is considered good and the waters can be classified as eutrophic, potentially evolving towards the hypertrophic conditions. The sanitary condition of the Brda River has improved from satisfactory to good in last years (Kowalkowski et al. 2006). Research conducted by Szkoda et al. (2014). showed that the average concentration of toxic elements (Pb, Cd, Hg, As) in free-living freshwater fish and water of the Brda River did not exceed permitted limits.

Absolute and relative fertility of spiny-cheek crayfish females from the Vistula Lagoon with total body length 8.1–11.6 cm was 535 and 17 eggs, respectively. Absolute and relative fertility was correlated with total body length and weight. Along with the increase in these parameters, the absolute fertility rose and the relative fertility decreased. Analyses indicated no statistically significant differences in absolute and relative fertility of spiny-cheek crayfish from the Vistula Lagoon in the comparisons with the representatives of this species from freshwater reservoirs (rivers, lakes). It may result from the fact that this species of crayfish is characterized by a strong adaptive properties in various reservoirs, with different water quality (the level of contaminations, and the degree of salinity and eutrophication). Its high reproductive and a tolerance to a wide range of environmental condition has enabled it to spread it far and wide.

In Poland, spiny cheek crayfish should be eliminated from the environment in accordance with the Regulation of the Minister of Agriculture and Rural Development of November 12, 2001 (Journal of Laws No. 138, item 1559), which requires that the captured individuals should not be returned to the tank. This regulation is a unique legal solution on a European scale. Because there is a misgiving that it may become a so-called “dead regulation” if it is not widespread enough, Authors of the manuscript are developing a ministerial project on the harvesting and management of crayfish as an addition to feed for exotic animals.
